# Application of TOPSIS method for flood susceptibility mapping using Excel and GIS

**DOI:** 10.1016/j.mex.2023.102263

**Published:** 2023-06-15

**Authors:** Rajib Mitra, Jayanta Das, Md. Kamruzzaman

**Affiliations:** a Department of Geography and Applied Geography, University of North Bengal, PO- North Bengal University, Darjeeling 734013, India; b Department of Geography, Rampurhat College, PO- Rampurhat, Birbhum 731224, India; c Institute of Bangladesh Studies, University of Rajshahi, Bangladesh

**Keywords:** TOPSIS, MCDM, Flood susceptibility mapping, GIS, Performance score, ArcGIS, TOPSIS

## Abstract

This study elaborately manifests a simplified Technique for Order Preference by Similarity to Ideal Solution (TOPSIS) multicriteria decision-making (MCDM) approach that goals to determine the disparity among the distances between the positive and negative ideal solutions. MCDM methods evaluate options based on a variety of criteria by using mathematical and analytical methodologies. This promotes a more transparent and objective decision-making process by removing human biases and subjective judgements. By considering the comparative proximity to the optimal situation, TOPSIS considers the distances between the ideal and the negative-ideal alternatives. This study has concentrated on the normalization process, the appropriate determination of the ideal and the anti-ideal solution, and the metric utilized to compute the euclidean distances from the ideal best and the ideal worst.

• This study expresses the simplified TOPSIS methodology as stated by Hwang and Yoon (1981). The categorization and weight assignments of the criteria have been executed from the expert's opinion and based on existing literatures.
• Integration of the TOPSIS technique with GIS has been properly performed for the production of a flood susceptibility map of a highly vulnerable region and visual interpretation of the TOPSIS algorithm.
• This kind of investigation saved time by sufficiently skilled specialized personnel in this field.

## Graphical abstract

**Figure:**
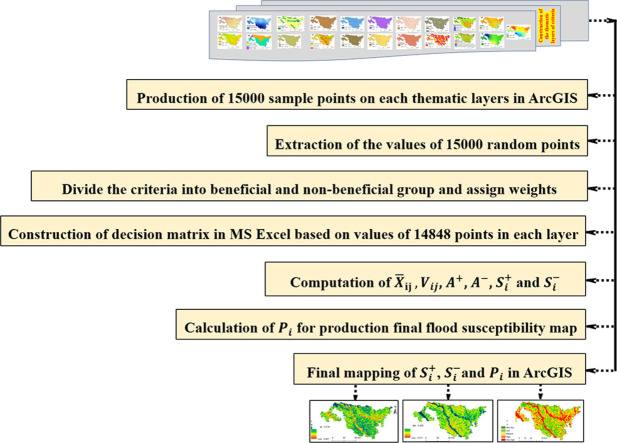

Specification table

**Table:** 

Subject area:	Hydrological modeling
More specific subject area:	Flood susceptibility modeling
Method name:	TOPSIS
Name and reference of original method:	Technique for Order Preference by Similarity to Ideal Solution by Hwang and Yoon (1981) Hwang, CL., Yoon, K. (1981). Methods for Multiple Attribute Decision Making. In: Multiple Attribute Decision Making. Lecture Notes in Economics and Mathematical Systems, vol 186. Springer, Berlin, Heidelberg. 10.1007/978–3–642–48,318–9_3.
Resource availability:	Microsoft Excel 2019 and ArcGIS (version 10.4.1) software

## Method details

### Background

Human beings generally aspire to make calculated decisions when faced with a choice between several options. Scientifically, the goal is to create numerical and analytical techniques that consider several options and a variety of variables. The Technique for Order Preference by Similarity to Ideal Solution method is shortly known as the TOPSIS method, which is a numerical technique for multicriteria decision-making (MCDM) analysis. It was first postulated by Hwang and Yoon (1981), which relies on determining the distances between every alternative and the ideal and anti-ideal solutions [1]. It is a simple technique with clear instructions that solves MCDM issues well. This strategy often focuses on the euclidean distance between the many decision-making options that are provided. According to this method, proximity is used to rank the alternative. It is regarded as one of the traditional MCDM techniques that have drawn a significant amount of attraction from both researchers and academics [2]. It's been employed efficiently in a variety of fields, viz., energy management, water resource management, environment management, human resource management, business, and marketing management. The TOPSIS MCDM approach has been selected for this study since it can find applications in a wide range of areas, analyze trade-offs, incorporate subjective judgements, give transparency, and enable data-driven settings. It provides decision-makers with an organized and efficient framework to solve complicated decision problems involving several factors. By employing TOPSIS, decision-makers can make informed and evidence-based decisions in identifying areas susceptible to flooding and implementing appropriate measures for prevention and mitigation. Several studies considered the integration of TOPSIS MCDM models with GIS, viz., Mitra and Das [3], Ramya and Devadas [4], Pathan et al. [5], Yildirim et al. [6].

### Steps to integrate the TOPSIS with GIS for the construction of FSM

**Fig. 1:**
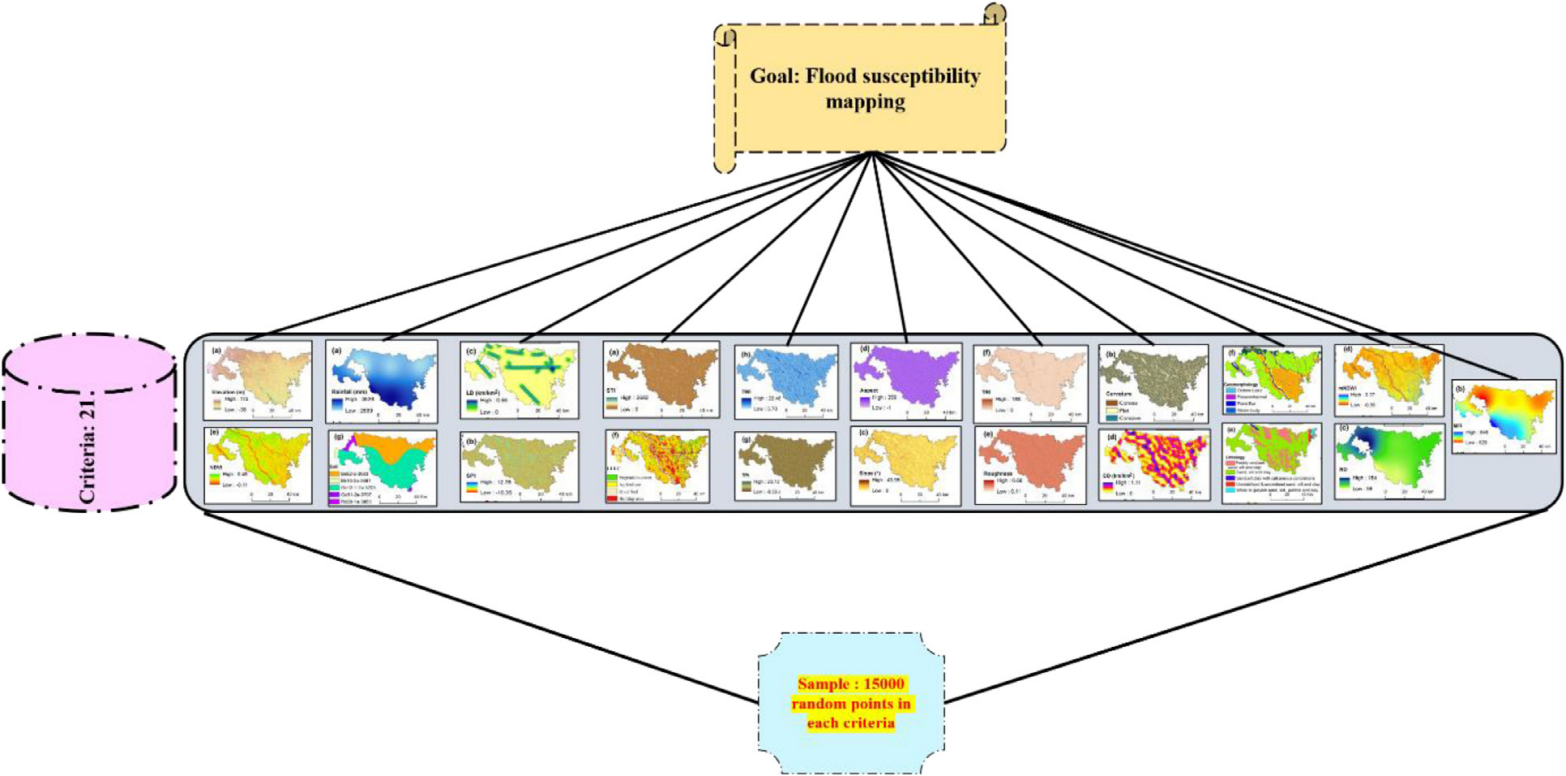
Hierarchical arrangements of the case study.

TOPSIS is not a pixel-based method; it is a discrete method. In the present day, it is necessary to integrate MCDM techniques with the GIS software. In this study, the integration has been conducted between the TOPSIS MCDM method and ArcGIS software to produce a flood susceptibility map (FSM). This methodological paper basically describes the overall steps utilized to produce the flood susceptibility map of the Koch Bihar district by the TOPSIS method in the published article by Mitra and Das (2022) [3]. The hierarchical representation of the flood susceptibility modeling using the TOPSIS MCDM model is manifested in Fig. 1. The description of all steps of the TOPSIS MCDM is given as follows:

**(i) Construction of the thematic layers of the FSM criteria**

In order to generate the theme layers in the ArcGIS software, this work first developed the spatial database of 21 flood triggering variables. After deriving the vector and raster data of 21 criteria, the thematic layers were prepared. A total of 20 thematic layers were taken into account after the multicollinearity issues were resolved, and all layers were then resampled at an equal spatial resolution.

**(ii) Extraction of the values from the thematic layers**

**Fig. 2:**
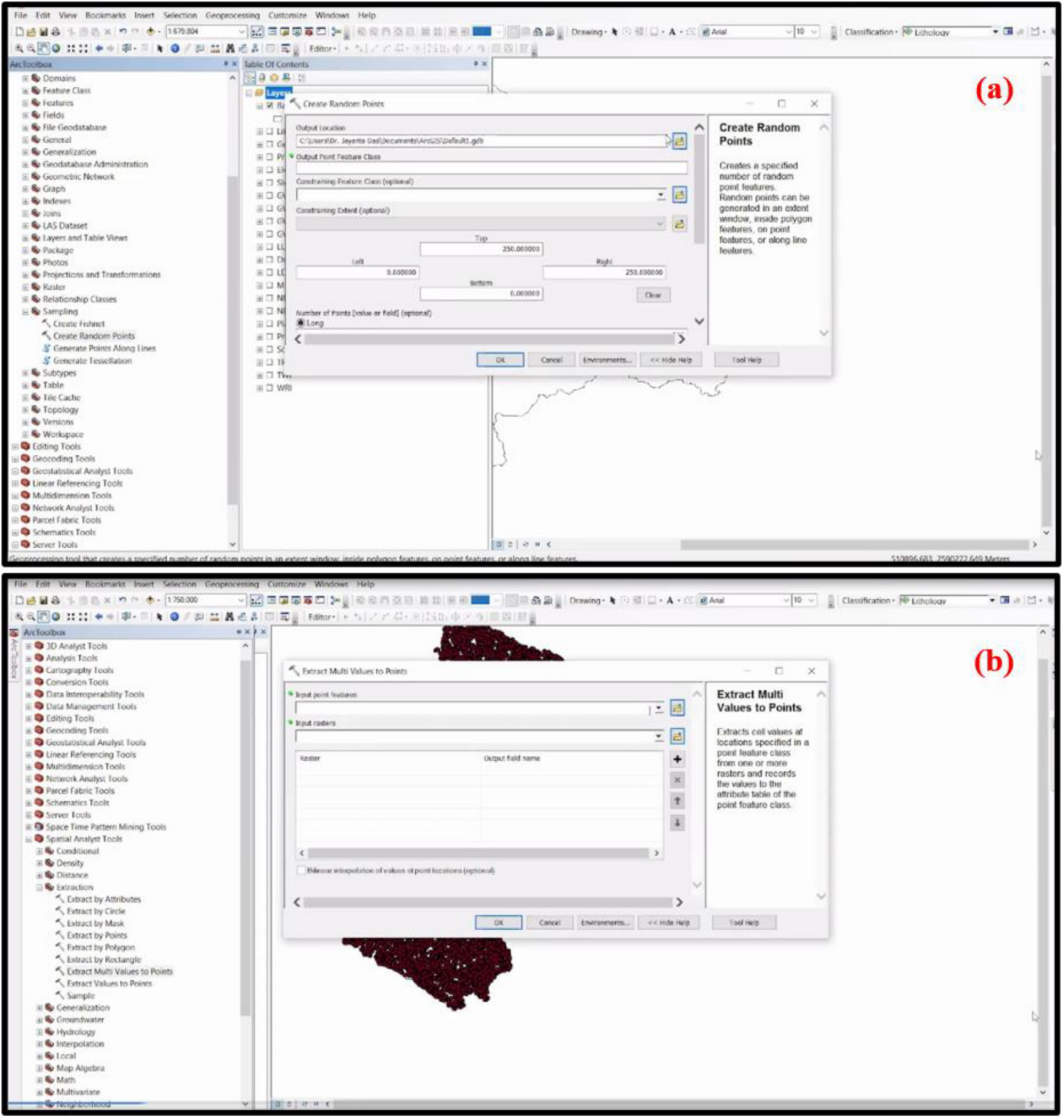
Procedure of **(a)** Creation of random points and **(b)** Extract Multi values to points in ArcGIS.

Then for the calculation of TOPSIS, random points were created in the 20 selected flood triggering variables for the extraction of the values. In this study, 15,000 random points were generated for the 20 criteria by employing the ‘Create Random Points' tool of Data Management Tools (Fig. 2a). Next, utilize the ‘Extract Multi Values to Points' feature of the Spatial Analyst Tools of the ArcGIS system to retrieve the data of these sample locations from all thematic layers (Fig. 2b). Additionally, using these extracted random points values of 15,000 sample points, Microsoft Excel 2019 was used to compute TOPSIS. Primarily, in this study, 15,000 sample points were taken into consideration, but there are observed some missing values of 152 random points and therefore removed these 152 random points. Further work was carried out with values of 14,848 random points.

**(iii) Identify the beneficial (B) and non-beneficial (NB) variables**

For the computation of TOPSIS, differentiation of the criteria is necessary as per their priority and contribution to generating the FSM for any region. In this case study, among the 20 criteria, 14 were identified as beneficial (B), and 6 were identified as non-beneficial (NB) criteria (Table 1). The B criteria are aspect, curvature, drainage density, lineament density, MFI, mNDWI TWI, lithology, LULC, geomorphology, soil, SPI, TPI, and rainfall deviation, whereas the NB criteria are elevation, roughness, slope, NDVI, STI, and TRI. It has been identified based on literature reviews and expert knowledge.

**Table 1:** Categorization of criteria.

Criteria	Criteria type

Aspect	Beneficial
Curvature	Beneficial
Drainage density	Beneficial
Lineament density	Beneficial
MFI	Beneficial
mNDWI	Beneficial
TWI	Beneficial
Lithology	Beneficial
LULC	Beneficial
Geomorphology	Beneficial
Soil	Beneficial
SPI	Beneficial
TPI	Beneficial
Rainfall deviation	Beneficial
Elevation	Non-Beneficial
Roughness	Non-Beneficial
Slope	Non-Beneficial
NDVI	Non-Beneficial
STI	Non-Beneficial
TRI	Non-Beneficial

**(iv) Weight assignments**

**Fig. 3:**
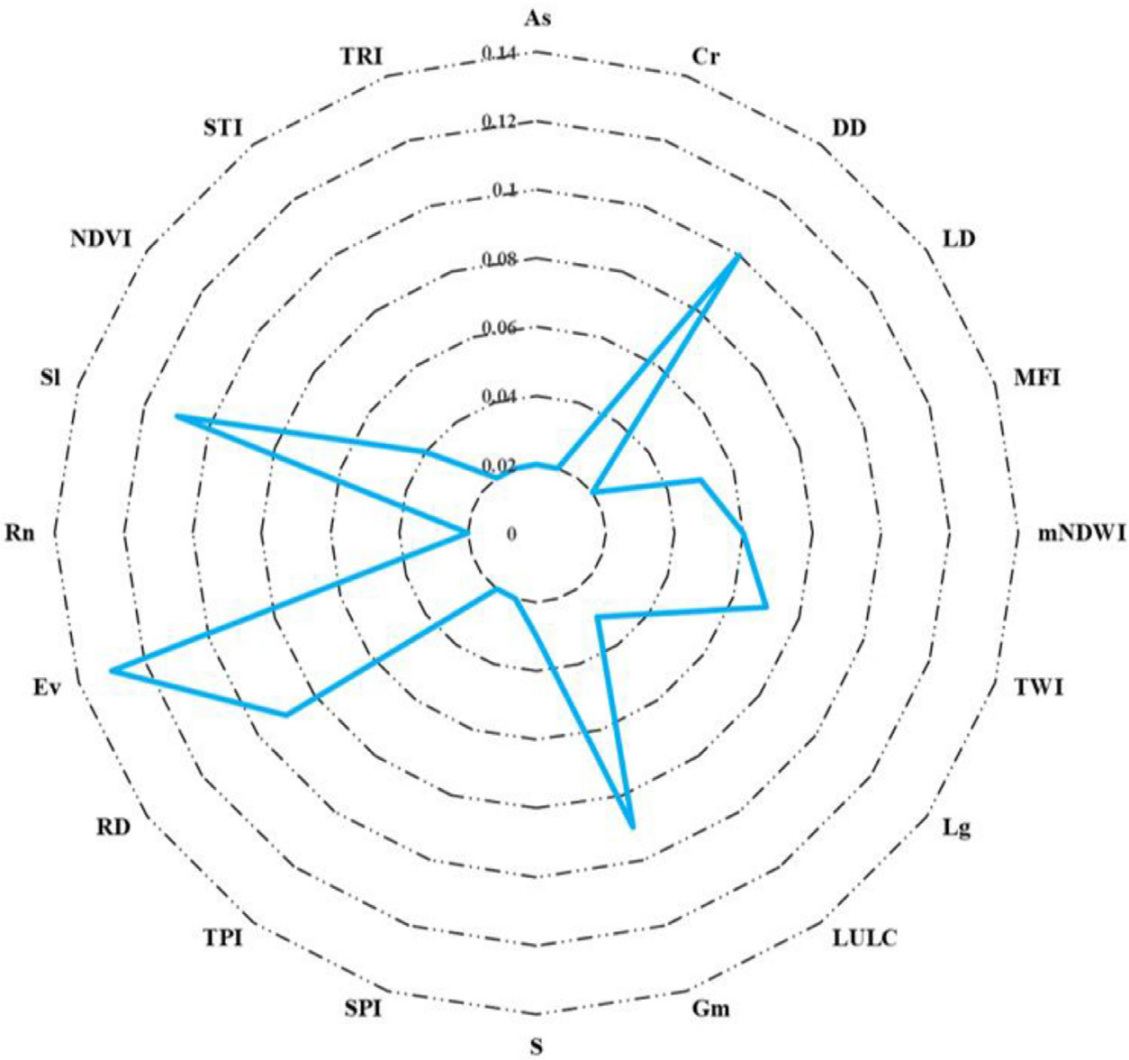
Assign weight for the criteria in the construction flood susceptibility map based on TOPSIS methodology.

A key step in the TOPSIS computation is the weight assignments of the criteria that cause floods. Thus, weights were attributed to this MCDM approach relying on analyst opinions from various studies. In this study, 70 percent weight was allocated for the eight criteria, i.e., elevation (13%), slope (11%), drainage density (10%), geomorphology (9%), rainfall deviation (9%), TWI (7%), mNDWI (6%), and MFI (5%). The weight of the other criteria, i.e., lithology, NDVI, LULC, soil, curvature, aspect, lineament density, STI, roughness, TRI, and TPI, are 4, 4, 3, 3, 2, 2, 2, 2, 2, 2, and 2 percent, respectively (Table 2). The associated weights of these indicators are shown in a radar diagram in Fig. 3.

**Table 2:** Weight assignments for the criteria.

Rank	Criteria	Weight (in percentage)

1	Elevation	13
2	Slope	11
3	Drainage density	10
4	Geomorphology	9
4	Rainfall deviation	9
5	TWI	7
6	mNDWI	6
7	MFI	5
8	Lithology	4
8	NDVI	4
9	LULC	3
9	Soil	3
10	Curvature	2
10	Lineament density	2
10	SPI	2
10	TPI	2
10	Roughness	2
10	STI	2
10	TRI	2
10	Aspect	2

**(v) Establishment of a decision matrix**

In the first stage, for calculation of the TOPSIS algorithm, must need to construct the decision matrix X, i.e., $X_{\text{ij}}$ . Eq. (1) illustrates the decision matrix with m alternatives and n attributes.

(1) $$Decision\;matrix=\begin{array}{c} \begin{array}{cccc} x_{1}\;\;\;\;\; & \;x_{2}\;\; & \;x_{j}\;\; & \;\;\;\;x_{n}\;\;\; \end{array}\\ \begin{array}{c} A_{1}\\ A_{2}\\ A_{i}\\ \vdots \\ A_{m} \end{array}\left[\begin{array}{cccccc} x_{11} & x_{12} & \cdots & x_{1j} & \cdots & x_{1n}\\ x_{21} & x_{22} & \cdots & x_{2j} & \cdots & x_{2n}\\ \vdots & \vdots & 0 & \vdots & 0 & \vdots \\ x_{i1} & x_{i2} & \cdots & x_{ij} & \cdots & x_{in}\\ \vdots & \vdots & 0 & \vdots & 0 & \vdots \\ x_{m1} & x_{m2} & \cdots & x_{mj} & \cdots & x_{mn} \end{array}\right] \end{array}$$

The table contains the exported values of 14,848 random points in each criterion representing the decision matrix of the present study as stated in Table 3 and Supplementary Material (SM) 1. The table of the decision matrix is also called the evaluation matrix of TOPSIS.

**Table 3:** Evaluation matrix of TOPSIS MCDM technique.

Sample No.	S1	S2	S3	S4	S5	…	S14844	S14845	S14846	S14847	S14848

Criteria											
Aspect	3	3	3	3	4	…	3	2	2	2	3
Curvature	1	1	1	3	1	…	1	1	2	1	1
Drainage density	0.41529	0.43748	0.40243	0.42236	0.45282	…	0.26449	0.28036	0.231151	0.10765	0.08852
Lineament density	0.12876	0.16901	0.75	0.75	0.20715	…	0	0	0	0	0
MFI	546.532	546.872	547.923	547.717	546.761	…	582.686	581.524	580.462	578.282	578.06
mNDWI	3	3	5	5	2	…	4	3	3	3	5
TWI	6.09906	6.30135	7.52064	5.94754	9.16537	…	9.08515	7.20265	14.2806	11.2454	6.55942
Lithology	3	3	3	3	3	…	3	3	3	3	3
LULC	2	1	5	5	2	…	2	4	4	4	4
Geomorphology	1	1	5	5	4	…	3	3	3	3	3
Soil	1	1	1	1	1	…	2	2	2	2	2
SPI	3	3	3	1	3	…	1	1	3	1	1
TPI	4	2	4	2	4	…	4	2	4	3	3
Rainfall deviation	159.456	159.259	158.425	158.557	159.153	…	123.171	123.85	124.5	126.064	126.201
Elevation	53	66	56	61	55	…	28	30	23	29	30
Roughness	0.45926	0.51515	0.53333	0.527778	0.55556	…	0.5	0.31746	0.62963	0.44444	0.51852
Slope	11.3129	9.28101	3.6832	4.43697	4.2646	…	0.96435	5.0559	0.681932	3.35445	2.40969
NDVI	0.27888	0.24841	0	0	0.21775	…	0.10782	0.17266	0.164163	0.18052	0.06491
STI	0.87955	0.68872	0.24942	0	0.7071	…	0	0	0.249416	0	0
TRI	16.3707	11.7473	4.69042	8.30662	7.07107	…	3.87298	7	6.55744	6.08276	5.38516

**(vi) Calculation of normalized matrix**

After that, the standardization of this decision matrix ($X_{\text{ij}}$) is made by vector normalization matrix, i.e., ${\bar{X}}_{\text{ij}\;}$. For the calculation of the normalized matrix following equation (Eq. (2)) is employed:

(2) $${\bar{X}}_{ij}=\frac{X_{ij}}{\sqrt{\sum_{i=1}^{n}X_{ij}^{2}}}$$

For the calculation of Eq. (2), $X_{\text{ij}}$ are the obtained 14,848 random points values, and using these values $X_{\text{ij}}^{2}$ was computed for all values (Table 4). All the obtained $X_{\text{ij}}^{2}$ values are summed by the SUM function of MS Excel and then square rooted by the SQRT function (Table 5). Thus, dividing the opted values of the numerator with the denominator assigned the values of the normalized vector as illustrated in Table 6 and SM 2.

**Table 4:** Calculation matrix of $X_{\text{ij}}^{2}.$

Sample No.	S1	S2	S3	S4	S5	…	S14844	S14845	S14846	S14847	S14848

Criteria											
Aspect	9	9	9	9	16	…	9	4	4	4	9
Curvature	1	1	1	9	1	…	1	1	4	1	1
Drainage density	0.172467	0.191384	0.161952	0.178388	0.205042	…	0.069954	0.078599	0.053431	0.011589	0.007835
Lineament density	0.016578	0.028565	0.5625	0.5625	0.04291	…	0	0	0	0	0
MFI	298,697.2	299,069	300,219.6	299,993.9	298,947.6	…	339,523	338,170.2	336,936.1	334,410.1	334,153.4
mNDWI	9	9	25	25	4	…	16	9	9	9	25
TWI	37.19853	39.70701	56.56003	35.37323	84.00401	…	82.53995	51.87817	203.9355	126.459	43.02599
Lithology	9	9	9	9	9	…	9	9	9	9	9
LULC	4	1	25	25	4	…	4	16	16	16	16
Geomorphology	1	1	25	25	16	…	9	9	9	9	9
Soil	1	1	1	1	1	…	4	4	4	4	4
SPI	9	9	9	1	9	…	1	1	9	1	1
TPI	16	4	16	4	16	…	16	4	16	9	9
Rainfall deviation	25,426.22	25,363.43	25,098.48	25,140.32	25,329.68	…	15,171.1	15,338.82	15,500.25	15,892.13	15,926.69
Elevation	2809	4356	3136	3721	3025	…	784	900	529	841	900
Roughness	0.210919	0.265381	0.284445	0.27855	0.308642	…	0.25	0.100781	0.396434	0.19753	0.268861
Slope	127.9817	86.13715	13.56596	19.6867	18.18681	…	0.929975	25.56212	0.465031	11.25233	5.806606
NDVI	0.077776	0.061709	0	0	0.047414	…	0.011624	0.02981	0.026949	0.032587	0.004213
STI	0.773605	0.474335	0.062208	0	0.499983	…	0	0	0.062208	0	0
TRI	267.9998	137.9991	22.00004	68.99994	50.00003	…	14.99997	49	43.00002	36.99997	28.99995

**Table 5:** $\sum_{i=1}^{n}X_{\text{ij}}^{2}$ and $\sqrt{\sum_{i=1}^{n}X_{\text{ij}}^{2}}$ values of 20 criteria.

$\sqrt{\sum_{i=1}^{n}X_{\text{ij}}^{2}}$	$\sqrt{\sum_{i=1}^{n}X_{\text{ij}}^{2}}$

127,696	357.34577
62,924	250.84657
2572.954926	50.724303
191.3480962	13.832863
4,771,605,448	69,076.808
119,591	345.81932
1,138,193.175	1066.8614
107,611	328.04116
121,280	348.25278
152,490	390.49968
44,080	209.95238
86,449	294.02211
154,491	393.05343
260,576,194.9	16,142.373
23,964,317	4895.3362
3808.440129	61.712561
213,305.371	461.84994
611.5960674	24.730468
4,806,373.276	2192.3442
1,498,611.559	1224.1779

**Table 6:** Calculation sheet of the normalized matrix of the TOPSIS.

Sample No.	S1	S2	S3	S4	S5	…	S14844	S14845	S14846	S14847	S14848

Criteria											
Aspect	0.008395	0.008395	0.008395	0.008395	0.011194	…	0.008395	0.005597	0.005597	0.005597	0.008395
Curvature	0.003987	0.003987	0.003987	0.01196	0.003987		0.003987	0.003987	0.007973	0.003987	0.003987
Drainage density	0.008187	0.008625	0.007934	0.008327	0.008927	…	0.005214	0.005527	0.004557	0.002122	0.001745
Lineament density	0.009334	0.012253	0.054371	0.054371	0.015017		0	0	0	0	0
MFI	0.007912	0.007917	0.007932	0.007929	0.007915	…	0.008435	0.008419	0.008403	0.008372	0.008368
mNDWI	0.008675	0.008675	0.014458	0.014458	0.005783		0.011567	0.008675	0.008675	0.008675	0.014458
TWI	0.005717	0.005906	0.007049	0.005575	0.008591	…	0.008516	0.006751	0.013386	0.010541	0.006148
Lithology	0.009145	0.009145	0.009145	0.009145	0.009145		0.009145	0.009145	0.009145	0.009145	0.009145
LULC	0.005743	0.002871	0.014357	0.014357	0.005743	…	0.005743	0.011486	0.011486	0.011486	0.011486
Geomorphology	0.002561	0.002561	0.012804	0.012804	0.010243		0.007682	0.007682	0.007682	0.007682	0.007682
Soil	0.004763	0.004763	0.004763	0.004763	0.004763	…	0.009526	0.009526	0.009526	0.009526	0.009526
SPI	0.010203	0.010203	0.010203	0.003401	0.010203		0.003401	0.003401	0.010203	0.003401	0.003401
TPI	0.010177	0.005088	0.010177	0.005088	0.010177	…	0.010177	0.005088	0.010177	0.007633	0.007633
Rainfall deviation	0.009878	0.009866	0.009814	0.009822	0.009859		0.00763	0.007672	0.007713	0.00781	0.007818
Elevation	0.010827	0.013482	0.011439	0.012461	0.011235	…	0.00572	0.006128	0.004698	0.005924	0.006128
Roughness	0.007442	0.008348	0.008642	0.008552	0.009002		0.008102	0.005144	0.010203	0.007202	0.008402
Slope	0.024495	0.020095	0.007975	0.009607	0.009234	…	0.002088	0.010947	0.001477	0.007263	0.005217
NDVI	0.011277	0.010045	0	0	0.008805		0.00436	0.006982	0.006638	0.007299	0.002625
STI	0.000401	0.000314	0.000114	0	0.000323	…	0	0	0.000114	0	0
TRI	0.013373	0.009596	0.003831	0.006785	0.005776		0.003164	0.005718	0.005357	0.004969	0.004399

**(vii) Calculation of weighted normalized matrix**

The formula for calculation of the weighted normalized matrix is described in Eq. (3) as follows:

(3) $$V_{ij}={\bar{X}}_{ij}\times W_{j}$$

where ${\bar{X}}_{ij\;}$ is the normalized vector and $W_{j}$ is the assigned weight for the 20 criteria. To obtain the values of weighted normalized matrix simple multiple, the values of ${\bar{X}}_{ij\;}$ and $W_{j}$ in MS Excel, as stated in Table 7 and SM 3.

**Table 7:** Calculation sheet of the weighted normalized matrix of the TOPSIS.

Sample No.	S1	S2	S3	S4	S5	…	S14844	S14845	S14846	S14847	S14848

Criteria											
Aspect	0.000168	0.000168	0.000168	0.000168	0.000224	…	0.000168	0.000112	0.000112	0.000112	0.000168
Curvature	7.97E-05	7.97E-05	7.97E-05	0.000239	7.97E-05		7.97E-05	7.97E-05	0.000159	7.97E-05	7.97E-05
Drainage density	0.000819	0.000862	0.000793	0.000833	0.000893	…	0.000521	0.000553	0.000456	0.000212	0.000175
Lineament density	0.000187	0.000245	0.001087	0.001087	0.0003		0	0	0	0	0
MFI	0.003956	0.003958	0.003966	0.003965	0.003958	…	0.004218	0.004209	0.004202	0.004186	0.004184
mNDWI	0.005205	0.005205	0.008675	0.008675	0.00347		0.00694	0.005205	0.005205	0.005205	0.008675
TWI	0.004002	0.004135	0.004935	0.003902	0.006014	…	0.005961	0.004726	0.00937	0.007378	0.004304
Lithology	0.003658	0.003658	0.003658	0.003658	0.003658		0.003658	0.003658	0.003658	0.003658	0.003658
LULC	0.001723	0.000861	0.004307	0.004307	0.001723	…	0.001723	0.003446	0.003446	0.003446	0.003446
Geomorphology	0.002305	0.002305	0.011524	0.011524	0.009219		0.006914	0.006914	0.006914	0.006914	0.006914
Soil	0.001429	0.001429	0.001429	0.001429	0.001429	…	0.002858	0.002858	0.002858	0.002858	0.002858
SPI	0.002041	0.002041	0.002041	0.00068	0.002041		0.00068	0.00068	0.002041	0.00068	0.00068
TPI	0.002035	0.001018	0.002035	0.001018	0.002035	…	0.002035	0.001018	0.002035	0.001527	0.001527
Rainfall deviation	0.00889	0.008879	0.008833	0.00884	0.008873		0.006867	0.006905	0.006941	0.007029	0.007036
Elevation	0.001407	0.001753	0.001487	0.00162	0.001461	…	0.000744	0.000797	0.000611	0.00077	0.000797
Roughness	0.000149	0.000167	0.000173	0.000171	0.00018		0.000162	0.000103	0.000204	0.000144	0.000168
Slope	0.002694	0.00221	0.000877	0.001057	0.001016	…	0.00023	0.001204	0.000162	0.000799	0.000574
NDVI	0.004511	0.004018	0	0	0.003522		0.001744	0.002793	0.002655	0.00292	0.00105
STI	8.02E-06	6.28E-06	2.28E-06	0	6.45E-06	…	0	0	2.28E-06	0	0
TRI	0.000267	0.000192	7.66E-05	0.000136	0.000116		6.33E-05	0.000114	0.000107	9.94E-05	8.8E-05

**(viii) Calculation of ideal best and ideal worst values**

The ideal best or positive ideal solution ($A^{+}$) and ideal worst or negative ideal solution ($A^{-}$) are calculated relying upon the values of weighted normalized, as stated below Eqs. (4) and (5):

$$A^{+}=\left\{V_{1}^{+},V_{2}^{+},.....,V_{j}^{+},.....,V_{n}^{+}\right\}$$

(4) $$A^{+}=\left\{\left(\underset{i}{MAX}V_{ij}|j\in \right),\left(\underset{i}{MIN}V_{ij}|j\in J_{2}\right)|\right\}$$

$$A^{-}=\left\{V_{1}^{-},V_{2}^{-},.....,V_{j}^{-},.....,V_{n}^{-}\right\}$$

(5) $$A^{-}=\left\{\left(\underset{i}{MIN}V_{ij}|j\in J_{1}\right),\left(\underset{i}{MAX}V_{ij}|j\in J_{2}\right)|\right\}$$

where, $J_{1}$ manifest the beneficial criteria and $J_{2}$ demonstrate the non-beneficial criteria.

It is an important task in TOPSIS MCDM to assign the values of ideal best and ideal worst. In this study, for beneficial criteria (14 criteria), calculated the ideal best value based on the maximum weighted normalized value among the 14,848 sample points weighted normalized values in each criterion using the ‘MAX' function in MS Excel, as manifested in Table 0. The ideal worst for these 14 beneficial criteria is the minimum weighted normalized value among all random point's weighted normalized values. On the other hand, for non-beneficial criteria (6 criteria), the minimum weighted normalized value among the 14,848 sample points in each criterion is the ideal best, which has been performed employing the ‘MIN' function in MS Excel.

The maximum weighted normalized value from 14,848 sample points is the ideal worst for NB criteria (Table 8).

**Table 8:** Ideal best (Zenith) and ideal worst (Nadir) values of 20 criteria.

**Criteria**	**Ideal best**$\left(A^{+}\right)$	**Ideal worst**$\left(A^{-}\right)$

Aspect	0.0002798	5.5968E-05
Curvature	0.0002392	7.973E-05
Drainage density	0.0021782	0
Lineament density	0.0010874	0
MFI	0.0046839	0.00376573
mNDWI	0.0086751	0.00173501
TWI	0.0146381	0.00288567
Lithology	0.0060968	0.00121936
LULC	0.0043072	0.00086144
Geomorphology	0.0115237	0.00230474
Soil	0.0042867	0.0014289
SPI	0.0034011	0.00068022
TPI	0.0025442	0.00050884
Rainfall deviation	0.0102562	0.00533349
Elevation	−0.000637	0.00217758
Roughness	3.601E-05	0.00028807
Slope	0	0.00583394
NDVI	−6.54E-05	0.0073109
STI	0	0.00914437
TRI	2.31E-05	0.00189811

**(ix) Calculation of euclidean distance from ideal best and euclidean distance from ideal worst**

**Fig. 4:**
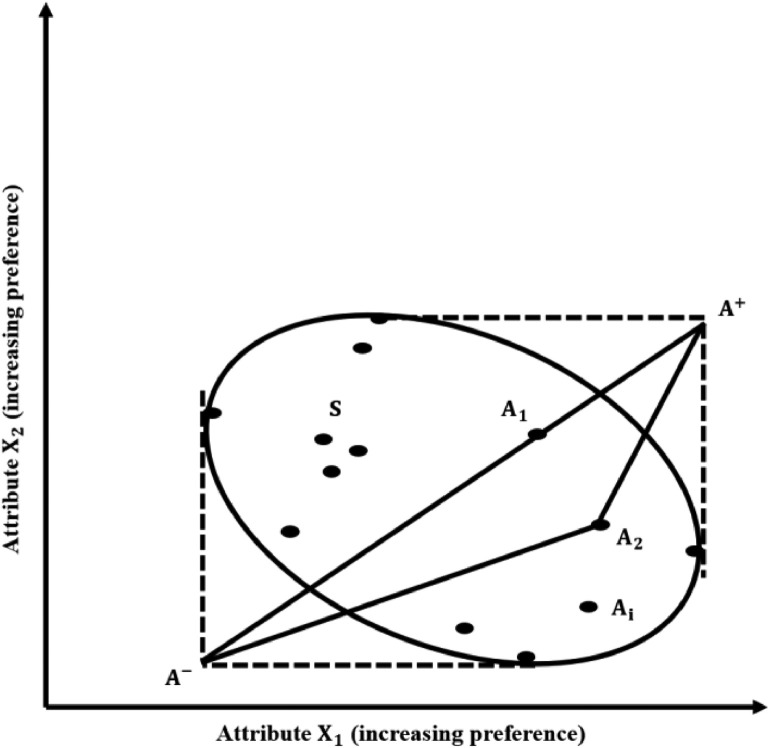
Diagrammatic representation of euclidean distance from ideal best and ideal worst (based on Hwang and Yoon, 1981).

The euclidean distance from ideal best $\left(S_{i}^{+}\right)$ and ideal worst $\left(S_{i}^{-}\right)$ are computed based on Eqs. (6) and (7):

(6) $$S_{i}^{+}=\sqrt{\sum_{j=1}^{n}{\left(V_{ij}-V_{j}^{\;+}\right)}^{2}}$$

(7) $$S_{i}^{-}=\sqrt{\sum_{j=1}^{n}{\left(V_{ij}-V_{j}^{\;-}\right)}^{2}}$$

where, $S_{i}^{+}$ is the euclidean distance from the ideal best, $S_{i}^{-}$ is the euclidean distance from the ideal worst, $V_{\text{ij}}$ is the value of the weighted normalized matrix, $V_{j}^{\;+}$ is the value of ideal best and $V_{j}^{\;-}$ is the value of the ideal worst. Based on Hwang and Yoon (1981), a diagrammatic depiction of the Euclidean distance from the ideal best and ideal worst is shown Fig. 4. Eqs. (6) and (7) were performed in MS Excel using several functions, and the result has been presented in Table 9 and SM 4.

**Table 9:** Euclidean distance from the ideal best and ideal worst.

Sample No.	$S_{i}^{+}$	$S_{i}^{-}$

S1	0.016437	0.011917
S2	0.016412	0.012069
S3	0.010967	0.018408
S4	0.012238	0.018161
S5	0.012345	0.014482
…	…	…
S14844	0.011927	0.014938
S14845	0.013285	0.013608
S14846	0.009786	0.0155
S14847	0.011399	0.014344
S14848	0.012813	0.015682

**(x) Calculation of performance score**

Determination of the performance score is the main task in TOPSIS MCDM for establishing the proximity to the ideal solution. The performance score has been assigned in this study based on Eq. (8).

(8) $$P_{i}=\frac{S_{i}^{-}}{S_{i}^{+}+S_{i}^{-}}$$

where, $P_{i}$ describe the performance score, $S_{i}^{-}$ represent the euclidean distance from the ideal worst and $S_{i}^{+}$ manifest the euclidean distance from the ideal best. After obtaining the values of $S_{i}^{-}$ and $S_{i}^{+}$, the $P_{i}$ has been calculated in MS Excel, as illustrated in Table 10 and SM 5.

**Table 10:** Performance score.

Sample No.	Pi

S1	0.420283
S2	0.423768
S3	0.626662
S4	0.597418
S5	0.539839
…	…
S14844	0.556046
S14845	0.506005
S14846	0.612994
S14847	0.557212
S14848	0.550341

**(xi) Ordering of the sequence of preference**

The alternatives are then ordered from best to worst (greater relative closeness number). The alternative that is ideal and the answer to the issue is at the beginning of the list.

**(xii) Mapping of the** $S_{i}^{+}$ **,** $S_{i}^{-}$ **and** $P_{i}$ **in GIS platform**

**Fig. 5:**
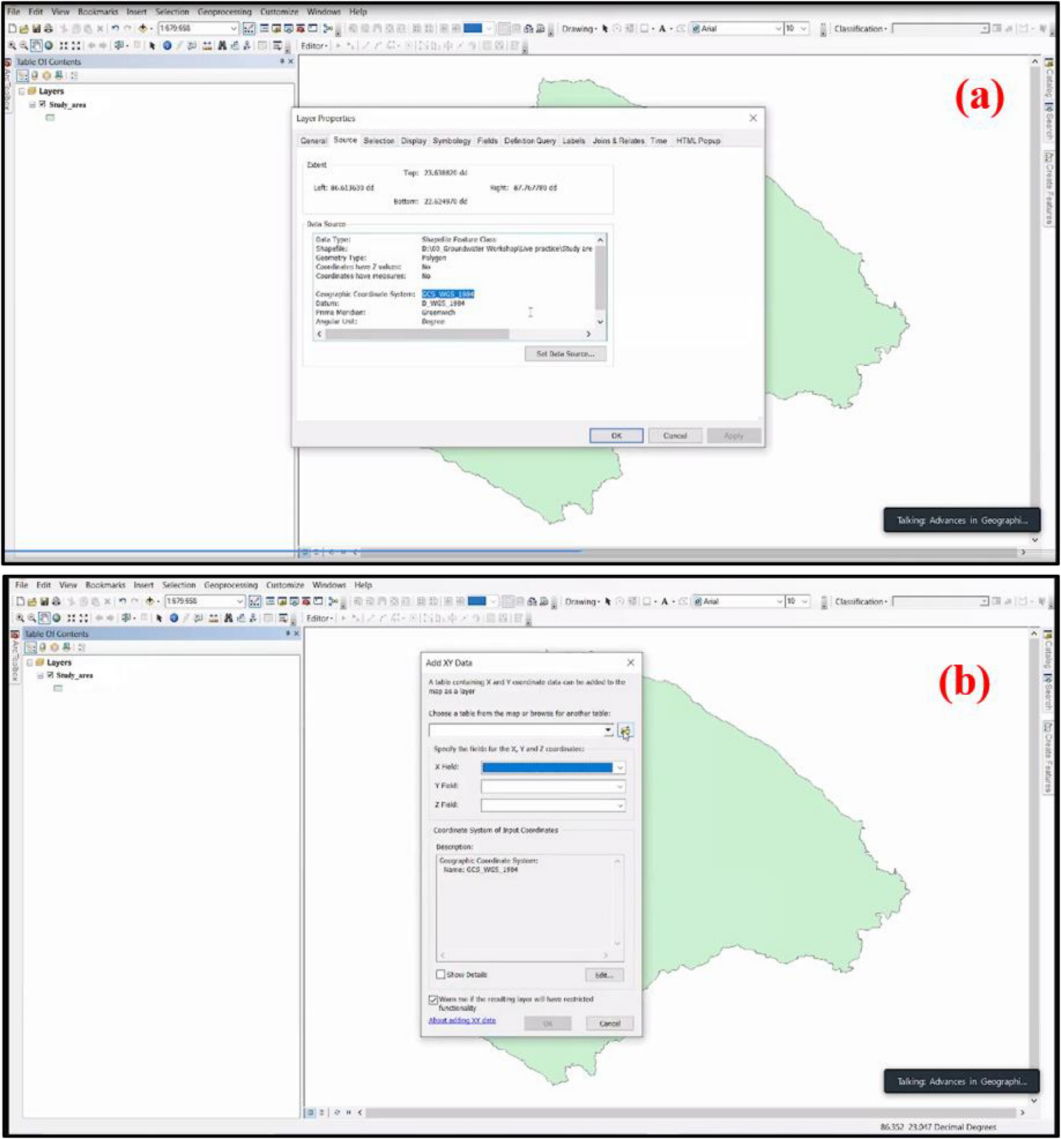
**(a)** Procedure to check the Geographic Coordinate System of the base map of the study area and **(b)** Addition of XY data from MS Excel to ArcGIS.
• The obtained values of $S_{i}^{+}$, $S_{i}^{-}$ and $P_{i}$ are extracted from MS Excel and added on ArcGIS from the ‘Add XY data' option in File Menu Bar (Fig. 5b). Then exported the data in ArcGIS to permanently save the XY data and further worked on it. When exporting the XY data, it is important that the study area map must have a Projected Coordinate System, i.e., WGS_1984_UTM_ZONE_45 N Projected Coordinate System had been utilized in the current research area (Fig. 6a).

After the establishment of three important algorithms (i.e., $S_{i}^{+}$, $S_{i}^{-}$ and $P_{i}$) of TOPSIS MCDM, it is the final task to map them to visualize the spatial differentiation of produced flood susceptibility map. For this purpose, the ArcGIS software is again employed. The procedure of map preparation is given as follows:

• Firstly, the Study area map is open in the ArcGIS platform, and it is necessary that the map have only a Geographic Coordinate System, i.e., CGS_WGS_1984 Geographic Coordinate System used for the present study area (Fig. 5a).

**Fig. 6:**
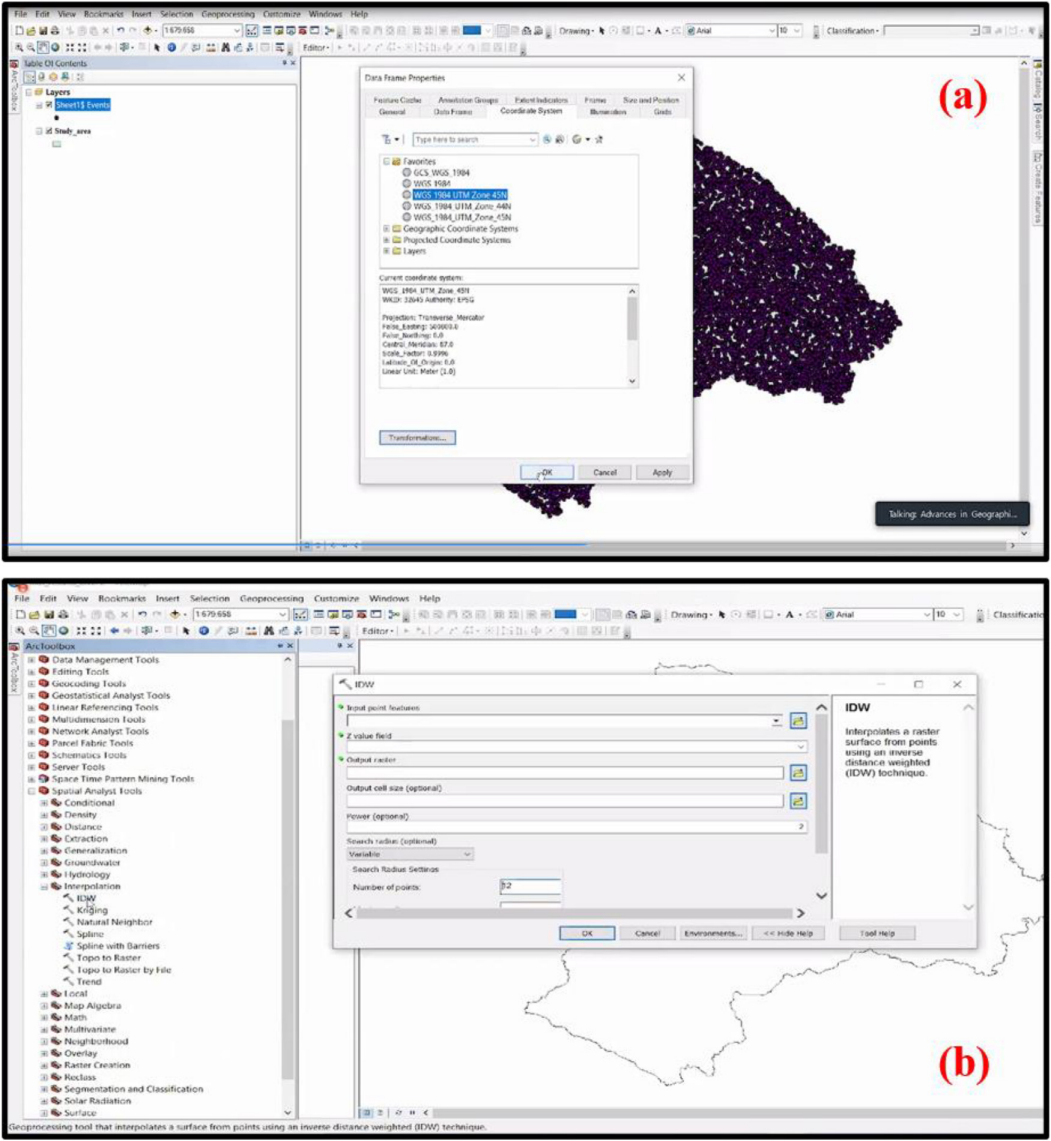
**(a)** Procedure to check the Projected Coordinate System of the base map of the study area and **(b)** Applying of Inverse Distance Weighting (‘IDW') tool in ArcGIS for mapping the $S_{i}^{+}$, $S_{i}^{-}$ and $P_{i}.$
• For mapping the criteria of positive effect ($S_{i}^{+}$), criteria of negative effect ($S_{i}^{-}$) and performance score ($P_{i}$), the interpolation technique is utilized. Therefore, the Inverse Distance Weighting (‘IDW') tool of Spatial Analyst Tools is employed in ArcGIS (Fig. 6b). In the ‘IDW' dialog box, after assigning the ‘Input point features', ‘Z value field', ‘Output raster', ‘Output cell size', and ‘Environment Settings', the maps of $S_{i}^{+}$, $S_{i}^{-}$ and $P_{i}$ have been produced. The map produced by the value $P_{i}$ is demonstrate the final flood susceptibility zonation map. In the article of Mitra and Das (2022), all the maps ($S_{i}^{+}$, $S_{i}^{-}$ and $P_{i}$) are manifested. Thus, the MCDM technique with GIS is integrated throughout the study.

**Fig. 7:**
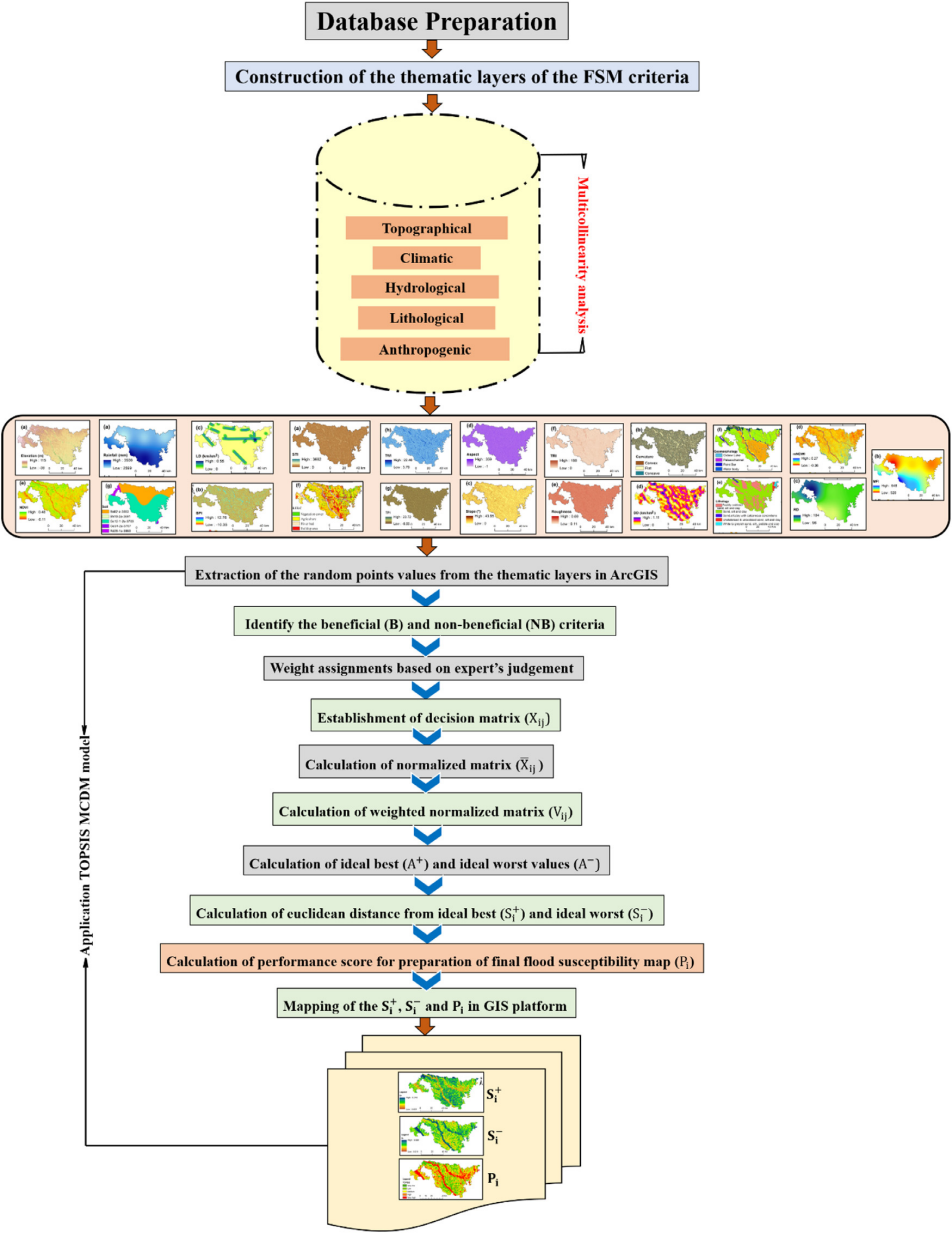
Methodological flowchart expressing overall steps of TOPSIS MCDM model with the integration of GIS for generation of the flood susceptibility map.

Methodological flowchart showing the fundamental processes used by the TOPSIS MCDM model with the integration of GIS to construct the flood susceptibility map in Fig. 7.

## Conclusions

The vulnerable and distressing conditions at a certain place can be initially assessed with the help of flood modeling. It has been challenging to model in developing nations because of a paucity of information and reliable calibration techniques. This investigation demonstrates the basic steps of the original TOPSIS MCDM technique using 21 criteria with 15,000 random points generation in each thematic layer, and then it integrates this model with GIS to visually interpret the flood susceptibility mapping of the Koch Bihar district of India. The study calibrates the model with all steps and, finally, the values of $S_{i}^{+}$, $S_{i}^{-}$ and $P_{i}$ of 14,848 points were computed, and lastly established, the ultimate ordering of the sequence of preference based on the relative distance of $P_{i}$. MS Excel 2019 and ArcGIS (10.4.1.) both the resources were simultaneously utilized for developing the model and then integrating it with GIS software.

Research on the detailed methodological description of MCDM models is limited globally. Specifically, an overall explanation of the integrated TOPSIS model with GIS is attempted throughout the present study for the first time, which demonstrates the novelty of this research.

TOPSIS is presently widely employed for modeling purposes in geographical and environmental studies. The original technique was adaptable sufficiently to enable numerous experiments and modifications. The expression of all the steps of integration will be helpful for academicians, engineers, and governmental agencies for the execution of the similar algorithm in other highly vulnerable places. The MCDM technique is crucial now as it facilitates complicated decision-making, encourages objectivity and stakeholder participation, tackles sustainability concerns, controls risk and uncertainty, optimizes resource allocation, and takes advantage of technological improvements.

## Declaration of Competing Interest

The authors declare that they have no known competing financial interests or personal relationships that could have appeared to influence the work reported in this paper.

## Appendix Supplementary materials

Image, application 1
